# Methyl Jasmonate Regulates Podophyllotoxin Accumulation in *Podophyllum hexandrum* by Altering the ROS-Responsive Podophyllotoxin Pathway Gene Expression Additionally through the Down Regulation of Few Interfering miRNAs

**DOI:** 10.3389/fpls.2017.00164

**Published:** 2017-02-14

**Authors:** Saptarshi Hazra, Dipto Bhattacharyya, Sharmila Chattopadhyay

**Affiliations:** Plant Biology Laboratory, Organic and Medicinal Chemistry Division, Council of Scientific and Industrial Research-Indian Institute Chemical BiologyKolkata, India

**Keywords:** mRNA decay, MeJA, miRNA, *Podophyllum hexandrum*, ptox, ROS

## Abstract

Podophylloxin (ptox), primarily obtained from *Podophyllum hexandrum*, is the precursor for semi-synthetic anticancer drugs viz. etoposide, etopophos, and teniposide. Previous studies established that methyl jasmonate (MeJA) treated cell culture of *P. hexandrum* accumulate ptox significantly. However, the molecular mechanism of MeJA induced ptox accumulation is yet to be explored. Here, we demonstrate that MeJA induces reactive oxygen species (ROS) production, which stimulates ptox accumulation significantly and up regulates three ROS-responsive ptox biosynthetic genes, namely, *PhCAD*3, *PhCAD*4 (cinnamyl alcohol dehydrogenase), and *NAC*3 by increasing their mRNA stability. Classic uncoupler of oxidative phosphorylation, carbonylcyanide *m*-chlorophenylhydrazone, as well as H_2_O_2_ treatment induced the ROS generation and consequently, enhanced the ptox production. However, when the ROS was inhibited with NADPH oxidase inhibitor diphenylene iodonium and Superoxide dismutase inhibitor diethyldithio-carbamic acid, the ROS inhibiting agent, the ptox production was decreased significantly. We also noted that, MeJA up regulated other ptox biosynthetic pathway genes which are not affected by the MeJA induced ROS. Further, these ROS non-responsive genes were controlled by MeJA through the down regulation of five secondary metabolites biosynthesis specific miRNAs viz. miR172i, miR035, miR1438, miR2275, and miR8291. Finally, this study suggested two possible mechanisms through which MeJA modulates the ptox biosynthesis: primarily by increasing the mRNA stability of ROS-responsive genes and secondly, by the up regulation of ROS non-responsive genes through the down regulation of some ROS non-responsive miRNAs.

## Introduction

The mechanism of action of ptox is based on inhibiting the polymerisation of tubulin and arresting of the cell cycle in the metaphase (Ayres and Loike, 1990; Buss and Waigh, 1995). Glycosidic derivatives of ptox, namely, etoposide, etopophos, and teniposide inhibit the cell cycle by inhibiting the DNA topo isomerase II (Fleming et al., 1989; Von Wartburg and Stahelin, 1993). Previous studies have also shown that ptox and its derivatives exhibit biological activity as strong antiviral agents and as antineoplastic drugs and its glycosidic derivatives, etoposide, etopophos (etoposide phosphate), teniposide are thus widely used for the treatment for various types of malignancy (Stahelin and von Wartburg, 1991; Canela et al., 2000). Ptox, is a polyphenolic substance, and formed by the combination of two phenylpropane units. Ptox is one of the most frequently used cytotoxic lignan isolated from podophyllin, which is an ethanolic extract of an endangered medicinal plant, *P. hexandrum* (Stahelin and von Wartburg, 1991; Gordaliza et al., 2004). This medicinal herb is generally grown at high altitude of the Himalayas and belongs to the Berberidaceae family. In one recent study, the *Podophyllum* Germination Network (PGN) has been constructed in reference to *Arabidopsis thaliana* to explore the underlying potential of seed germination mechanisms (Dogra et al., 2015). Previous studies reported that the chemical synthesis of cyclolignan like ptox is yet to be feasible. Thus, various biotechnological techniques like biotransformations, etc. (Kutney, 1999), especially transgenic hairy roots produced by infection of plants with *Agrobacterium rhizogenes* and cell culture based production of cyclolignan like ptox have been studied extensively (Kadkade, 1981; van Uden et al., 1989; Heyenga et al., 1990; Seidel et al., 2002; Chattopadhyay et al., 2003). It was also reported that MeJA treatment induce the production of lariciresinol, another lignan, in hairy root cultures of *Isatis indigotica* (Chen et al., 2015).

Previously, the cell suspension cultures of *Linum* spp. was noted with higher yield of ptox using MeJA and salicylic acid (SA) as elicitors (van Furden et al., 2005; Yousefzadi et al., 2010). MeJA treatment also induced the other secondary metabolie production like paclitaxel in the cell culture of *Taxus cuspidate* (Lenka et al., 2015). Treatment of the cell culture of *L. nodiflorum* with coronalon, indanoyl-isoleucine and MeJA induced the accumulation of 6-methoxypodophyllotoxin, a lignan related to ptox (Berim et al., 2005). In our previous studies, we have also reported the 8- to 10-fold higher yield of ptox in MeJA treated old cell culture of *P. hexandrum* (Bhattacharyya et al., 2012).

Jasmonic acid (JA) and its methyl derivative, MeJA, are well known as plant signaling compound that involves in stress management and development. Wang and Wu (2005) reported that exogenous treatment of JA and MeJA, actually mimic the effects of wounding and these elicitors bring the relative response like production of secondary metabolite as well as ROS. It has also been reported that MeJA induced ROS alter the mitochondrial dynamics that resulted in photosynthetic dysfunction and cell death (Zhang and Xing, 2008). According to Wang and Wu (2005) MeJA induces the ROS, which includes H_2_O_2_ and NO, in cell culture of *Taxus* sp. and the introduction of ROS inhibitor to culture reduce the MeJA induced taxol production. Similar to the changes in taxol from *Taxus sp.*, concentration of other secondary metabolites like phytoalexin were also increased by ROS via cyclopentenone isoprostanes in tomato (Thoma et al., 2003). MeJA induced production of H_2_O_2_ also reported in rice leaves. According to Orozco-Cárdenas et al. (2001) H_2_O_2_ acts as a second messenger upon treatment with MeJA and induced systemin production to the tomato plant.

Previously, we also did a whole transcriptome analysis of control and MeJA treated cell culture of *P. hexandrum* and identified ptox specific CAD isoforms namely, PhCAD3 and PhCAD4 (Bhattacharyya et al., 2016, 2013). However, the mechanism through which MeJA regulates ptox biosynthetic pathway genes is yet to be understood. This study revealed the molecular mechanism of MeJA altered ptox accumulation by regulating ptox biosynthetic pathway genes at transcript level. This has shown that MeJA induced ROS alters the mRNA stability of selected pathway genes to regulate their expression at transcript level. In addition to that, it was also noted that MeJA up regulates other ptox biosynthetic pathway genes which were not regulated by ROS. These ROS non-responsive genes may be regulated by the down regulation of particular MeJA responsive miRNAs, which are interfering with ptox pathway genes. Together, this study suggests two possible molecular mechanisms of MeJA induced ptox accumulation in *P. hexandrum* cell culture.

## Materials and Methods

### Plant Growth Condition and Treatment

Callus was derived from mature leaves of *P. hexandrum* in Murasige and Skoog (MS) medium supplemented with 2.68 μM alpha-Naphthaleneacetic acid (NAA) and 8.88 μM 6-Benzylaminopurine (BAP; Chakraborty et al., 2010) Subculture of callus was done after every 2 weeks in the above mentioned medium. Cell suspension culture was initiated according to Bhattacharyya et al. (2012). In brief, 5 g cells of fresh green callus was inoculated in 50 ml culture containing 60 mM total N_2_ content, 1.25 mM potassium dihydrogen phosphate, 6% glucose and 11.41 μM 3-Indoleacetic acid (IAA). Cultures were incubated at 22°C for 3 days at 110 rpm before treatment. Three days old cell cultures were treated with MeJA (100 μM); classic uncoupler of oxidative phosphorylation CCCP (Izeradjene et al., 2005; 100 μM); ROS inhibitor, DPI (1 μM); ROS inhibitor, DETC (10 μM), (Sigma-Aldrich, USA) and H_2_O_2_ (Merck, USA; 20 mM) in different combination. Two hours was noted as an optimum time point for harvesting the cells after treatment.

### Isolation of Protoplast for ROS Determination by Confocal and Flow Cytometry

Protoplast was isolated from 200 mg cells from 3 days old cell suspension culture (Zhang and Xing, 2008). Isolated protoplasts were finally washed in 0.35 M mannitol in liquid MS medium (pH 5.8) and treated with 100 μM MeJA or with ROS inducers or ROS inhibitors as designed and sampled after 2 h for confocal and FACS study.

### Determination of ROS by Confocal Microscopy and FACS

For confocal microscopy treated protoplast was incubated with 6-carboxy-2,7-dichlorodihydrofluorescein diacetate (DCFDA; Sigma-Aldrich; 5 mM) in the dark for 5 min (Zhang and Xing, 2008). The intracellular ROS production was visualized under the Andor Spinning Disk Confocal Microscope. For DCFDA, solid state laser excitation was 488 nm and emission was at 525 nm. For ROS generation analysis by FACS, protoplasts were incubated with 40 μM DCFDA for 5 min in the dark after the treatment. DCFDA is deacetylated by intracellular esterase, which is further oxidized by ROS to the fluorescent compound 2,7-dichlorofluorescein (DCF). DCF fluorescence was detected by FACS (Becton–Dickinson), using Cell Quest software. Ten thousand events were recorded for each sample.

### Extraction of Ptox by HPLC Analysis

The ptox extraction from various samples was performed as described previously (Bhattacharyya et al., 2012). In brief, 100 mg of harvested cells was homogenized with liquid nitrogen and then extracted with ethyl acetate for over night. For HPLC analysis, the supernatant was decanted after ethyl acetate extraction, evaporated to dryness and dissolved in methanol for further use (Kartal et al., 2004). Column and instrumentation for analysis were performed as standardized (Bhattacharyya et al., 2012).

### Extraction of RNA and Quantitative RT-PCR (RT-qPCR) Analysis

Total RNA was extracted from the harvested cells from control and treated culture with Trizol (Invitrogen) according to a standardized protocol (Bhattacharyya et al., 2016) in three replicates. cDNA was synthesized using RevertAid H Minus First Strand cDNA Synthesis kit (Fermentas, USA). The qRT-PCR was performed using the Light Cycler 96 System (Roche Applied Science, USA) with FastStart Essential DNA Green Master (Roche Applied Science). The constitutively expressed actin gene was used as the reference gene. Gene specific primers for performing RT-qPCR are given in Supplemental Table 1. Total 19 ptox biosynthetic pathway genes were selected, including five phenylpropanoid pathway related transcription factors and grouped them in four groups as mentioned below. Group A: four CAD isoforms namely *PhCAD*1, *PhCAD*2, *PhCAD*3, *PhCAD*4; Group B: five genes upstream of CAD; Group C: five genes downstream of CAD and Group D: five transcription factors related to phenylpropanoid pathway (All genes were submitted under the accession numbers SRX180871 and SRX180389). Here, CAD isoforms were kept in the middle position because CAD is the most important rate limiting gene, which actually controls the trafficking of coniferyl alcohol, a precursor of ptox and lignin biosynthesis, toward ptox and lignin.

### mRNA Stability Assay

mRNA stability assay was performed according to Datta et al. (2015). In brief, the control and treated cell cultures were further treated with 200 mM actinomycin D (Sigma-Aldrich) and cells were harvested after 2, 4, 6, 8, 10, and 12 h. Total RNA was isolated for further RT-qPCR by using gene specific primers for *PhCAD*3, *PhCAD*4, and *NAC*3. Cell culture treated with water was taken as mock treated. Prior to the treatment with water, cultures were pre-incubated in actinomycin D for 30 min to allow proper distribution of the antibiotic.

### RT-qPCR of miRNA

Stem loop RT-qPCR of miRNA was done according to Varkonyi-Gasic et al. (2007) with minor modifications. In brief, the total miRNA was isolated from the harvested cells of the treated and control cell culture of *P. hexandrum* using the mirPremier microRNA isolation kit (Sigma-Aldrich) according to the manufacturer’s instructions. Hundred nano-gram miRNA was used for cDNA preparation using stem loop RT primers according to Biswas et al. (2016). The RT-qPCR was performed using Light Cycler 96 System (Roche Applied Science) with FastStart Essential DNA Green Master (Roche Applied Science). The constitutively expressed *ubiquitin*-6 gene was used as the reference gene. miRNA specific primers for performing RT-qPCR are listed in Supplemental Table 2.

## Result

### Detection of ROS and Measurement Ptox Content From Treated *P. hexandrum* Cell Culture

**FIGURE 1:**
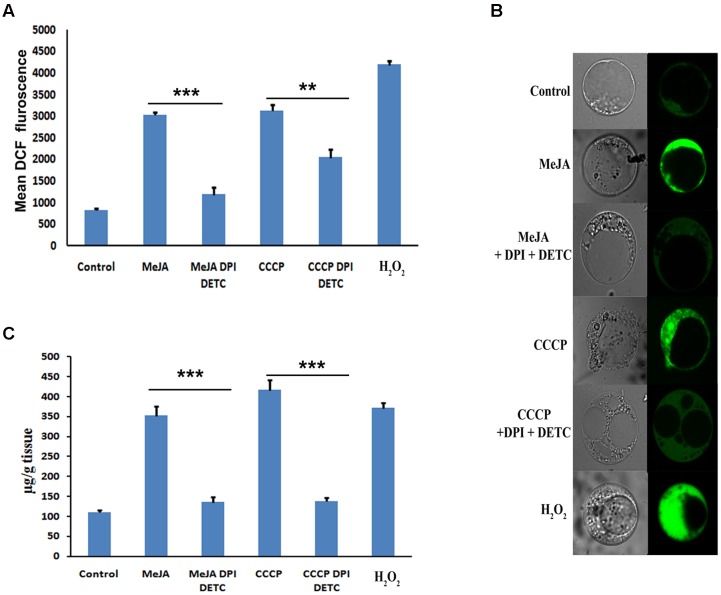
**(A)** Content of ptox after various treatments to the cell culture. **(B)** Measurement of ROS after various treatments to the cell culture by FACS. **(C)** Detection of ROS by confocal microscopy after staining with DCFDA of various treated cultures. The statistical analysis of the difference between fold changes was done using GraphPad Instat-3 software. Comparison between groups was done using one-way analysis of variance (ANOVA) followed by Student–Newman–Keuls test. Data were fitted using Sigma plot represented as means ± SD. *p* < 0.05 was accepted as level of significance; ^∗∗∗^ highly significant *p* < 0.001; ^∗∗^ significant *p* < 0.01; ^∗^ less significant *p* < 0.05; NS not significant for *p* > 0.05.

The detection of ROS is concurrent to the enhanced ptox accumulation in 2 h MeJA treated 3 days old *P. hexandrum* cell culture (**Figures 1A–C**). Treatment with CCCP and H_2_O_2_ also induced the ROS in the cell culture after 2 h of treatment. Amount of ROS produced after H_2_O_2_ treatment was maximum (**Figures 1A,B**). The independent treatments of both CCCP and H_2_O_2_ increased the ptox content in the cell culture, where the maximum amount of ptox was accumulated after CCCP treatment for 2 h (**Figure 1C**). Interestingly, when ROS production was inhibited by the combination of DPI and DETC treatment in MeJA and CCCP treated cultures, decreased ptox content was noted as well (**Figures 1A–C**).

### Transcript Assay of Selected Ptox Biosynthetic Pathway Genes After Independent MeJa and the Combination of DPI, DETC, and MeJA Treatment

**FIGURE 2:**
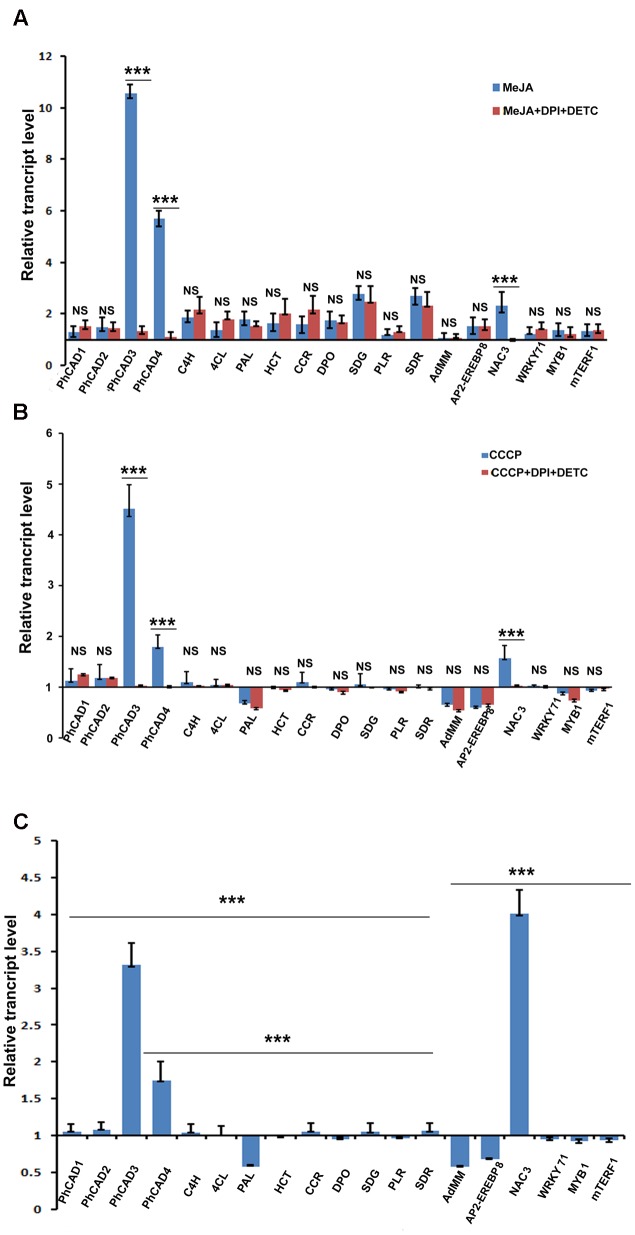
**Transcript assay of 19 ptox biosynthetic pathway genes after various treatments. (A)** Expression pattern after MeJA and MeJA+DPI+DETC ttreatment. **(B)** Expression pattern after CCCP and CCCP+DPI+DETC treatment. **(C)** Expression pattern after H_2_O_2_ treatment. The statistical analysis of the difference between fold changes was done using GraphPad Instat-3 software. Comparison between groups was done using one-way analysis of variance (ANOVA) followed by Student–Newman–Keuls test. Data were fitted using Sigma plot represented as means ± SD. *p* < 0.05 was accepted as level of significance; ^∗∗∗^ highly significant *p* < 0.001; ^∗∗^ significant *p* < 0.01; ^∗^ less significant *p* < 0.05; NS not significant for *p* > 0.05.

To understand how MeJA induced ROS generation affected the ptox biosynthesis consequently increasing the ptox content, the expression pattern of 19 ptox biosynthetic pathway related genes was checked, in two sets of experiments viz. the first set of treatment was with only MeJA and the second set was a combination of MeJA, DPI, and DETC. Independent MeJA treatment up regulates all 19 selected ptox biosynthetic pathway genes, including five transcription factors related to ptox biosynthesis. Amongst these, *PhCAD*3 was significantly up regulated, followed by *PhCAD*4*, C*4*H, SDR, SDG, DPO, NAC*3, and so on. Other transcripts like *PLR, AdMM*, and *mTERF*1 were comparatively less up regulated after the treatment. Interestingly, when ROS was inhibited in the second set, viz. the combination treatment of MeJA, DPI and DETC, up regulation of only three genes, namely *PhCAD*3, *PhCAD*4, and *NAC*3 were decreased significantly (**Figure 2A**). Meanwhile, other 16 genes remained unaltered and exhibited almost same expression pattern like independent MeJA treatment (**Figure 2A**).

### Transcript Assay of Selected Ptox Biosynthetic Pathway Genes After Independent CCCP, H_2_O_2_, and the Combined DPI, DETC, and CCCP Treatment

To further confirm the ROS-responsiveness of *PhCAD*3, *PhCAD*4, and *NAC*3, *P. hexandrum* culture was treated independently with CCCP and H_2_O_2_ as well as with a combination treatment of CCCP, DPI, and DETC. Only three genes, namely, *PhCAD*3, *PhCAD*4, and *NAC*3 were significantly up regulated after CCCP and H_2_O_2_ treatments. All other 16 genes were remained unaffected, although sometimes insignificant down regulation was noted. However, the genes like *AdMM*, *PAL*, and *AP*2-*EREBP*8 were down regulated insignificantly. While, the ROS in CCCP treated culture was inhibited with the combination treatment of DPI and DETC, only *PhCAD*3, *PhCAD*4, and *NAC*3 were affected and their up regulation was decreased significantly (**Figure 2B**). However, for other 16 genes the results were similar to those obtained in the case of CCCP treatment alone (**Figure 2B**). Again, the H_2_O_2_ treatment of the cell culture revealed significant up regulation of only *PhCAD*3*, PhCAD*4, and *NAC*3 genes, however, *AP*2-*EREBP*8, *AdMM*, and *PAL* were down regulated after the treatment (**Figure 2C**). All other selected genes remain unaffected after the treatment.

### mRNA Stability Assay of *PhCAD*3, *PhCAD*4, and *NAC*3

**FIGURE 3:**
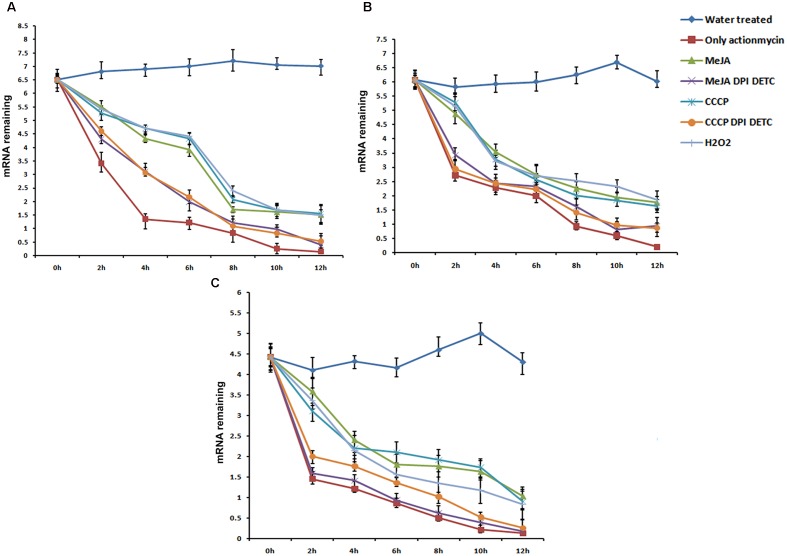
**mRNA Stability assay of three ROS responsive genes viz, *PhCAD*3, *PhCAD*4, and *NAC*3 after various treatments. (A)** mRNA stability of *PhCAD*3. **(B)** mRNA stability of *PhCAD*4. **(C)** mRNA stability of *NAC*3. Data are the mean ± SD for three individual experiments.

mRNA stability level of 3 ROS-responsive genes, *PhCAD*3, *PhCAD*4, and *NAC*3 was checked after independent treatment with MeJA, CCCP, H_2_O_2_ and the combination treatment of MeJA+DPI and DETC and CCCP +DPI and DETC. Three days old cell culture was mock treated with water and considered as a control. Cell treated with actinomycin D alone revealed that remaining mRNA significantly reduced after 4 h of actinomycin D treatment for *PhCAD*3 whereas for *PhCAD*4 and *NAC*3 it significantly reduced after 2 h of treatment. For all three genes after 12 h of treatment there was almost no remaining mRNA (**Figure 3**). Hence, we check the mRNA stability for all three genes up to 12 h after the respective treatments. MeJA treatment resulted in the higher transcript level of *PhCAD*3, *PhCAD*4, and *NAC*3 after actinomycin D treatment which indicated that MeJA increased the mRNA stability of *PhCAD*3, *PhCAD*4, and *NAC*3 up to 1.61-, 1.9-, and 2.46-fold, respectively, after 2 h. In two separate, independent treatment with CCCP and H_2_O_2_ induced ROS, which in turn increased the mRNA stability of *PhCAD*3, *PhCAD*4, and *NAC*3. But when ROS was inhibited with DPI and DETC in MeJA and CCCP treated cultures, then mRNA stability of *PhCAD*3, *PhCAD*4, and *NAC*3 were decreased significantly.

### miRNA Transcript Assay after Various Treatments

**FIGURE 4:**
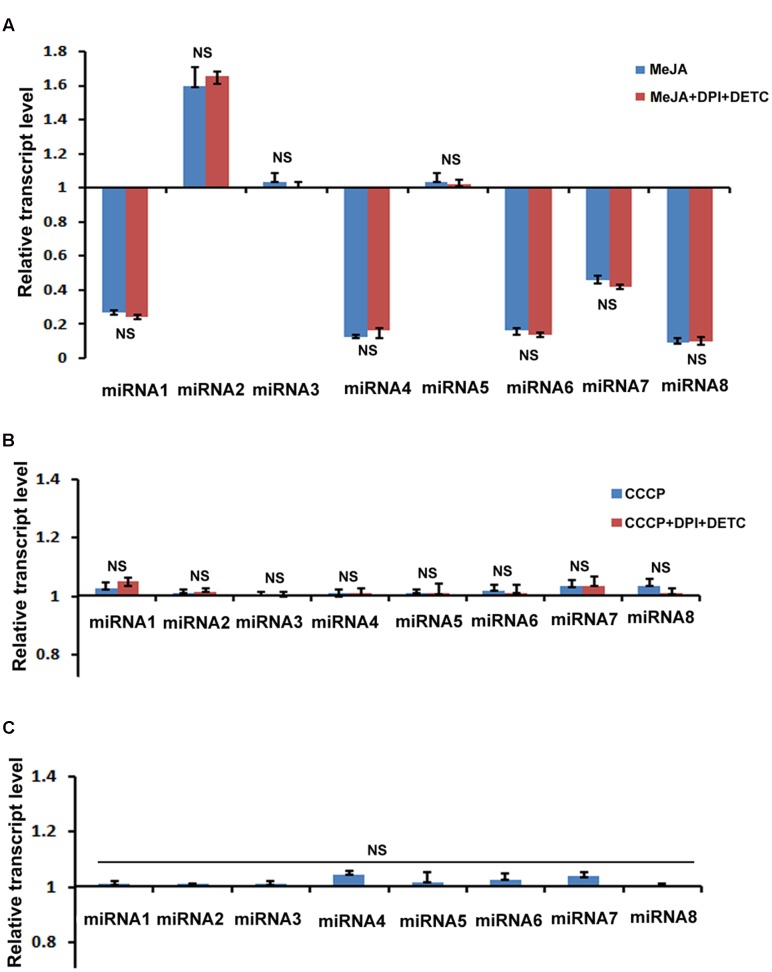
**Effect of MeJA and other ROS generating compound’s treatment on the expression of eight selected miRNAs. (A)** RT-qPCR analysis of MeJA and MeJA+DPI+DETC treatment. **(B)** RT-qPCR analysis after CCCP and CCCP+DPI+DETC treatment. **(C)** RT-qPCR analysis after H_2_O_2_ treatment. The statistical analysis of the difference between fold changes was done using GraphPad Instat-3 software. Comparison between groups was done using one-way analysis of variance (ANOVA) followed by Student–Newman–Keuls test. Data were fitted using Sigma plot represented as means ± SD. *p* < 0.05 was accepted as a level of significance; ^∗∗∗^ highly significant *p* < 0.001; ^∗∗^ significant *p* < 0.01; ^∗^ less significant *p* < 0.05; NS not significant for *p* > 0.05. miRNA1, miR172i; miRNA2, miR1873; miRNA3, miR5538; miRNA4, miR035; miRNA5, miR5532; miRNA6, miR1438; miRNA7, miR2275; miRNA8, miR8291.

We were also interested to explore the mechanism of MeJA mediated up regulation of upstream and downstream genes of *PhCAD*s which are not regulated by MeJA produced ROS. It was well established that miRNAs play a vital role as a *trans*-acting regulator of gene expression (Carlborg and Haley, 2004). Here, we selected eight miRNAs from our previously published whole transcriptome data (Bhattacharyya et al., 2013) after KEGG pathway analysis. These eight selected miRNAs were specifically targeted to the secondary metabolite pathway specially phenylpropanoid pathway. Only five miRNAs namely miR172i, miR035, miR1438, miR2275, miR8291 were significantly down regulated after MeJA treatment (**Figure 4A**). But all these eight miRNAs were completely non-responsive to CCCP and H_2_O_2_ induced ROS (**Figures 4B,C**). Furthermore, these miRNAs also noted with any effect when ROS was inhibited by combining DPI and DETC in the MeJA and CCCP treated cultures (**Figures 4A,B**). These five miRNAs are interfering with the target gene expression, namely, four *coumarate ligage* (4*CL*), *Cytochrome P*450, *Caffeoyl–Co*A *methyltransferase*, *Cytochrome P*450, and *Chalclone synthase* and the decline in the level of these miRNAs may bring about an up-regulation of the target genes.

## Discussion

It is well established that MeJA is a signal molecule in plant defense responses and an effective inducer of secondary metabolite accumulation in plant cell cultures by means of ROS as a secondary signaling molecule. According to You and Chan (2015) during stress condition ROS plays a vital role in the regulation of complex signaling network of plant stress response. It was also reported by Wang and Wu, 2005 that MeJA also induced the production of H_2_O_2_, intracellular malondialdehyde content and lipoxygenase. ROS also regulates the gene expression, translation, metabolism, and turnover (Singh et al., 2016). Exogenous application of MeJA also alters the stomatal closure by ROS where ROS inhibitor DPI prohibited the stomatal closure (Suhita et al., 2004). In this study, we explore the mechanism through which MeJA affects the increased accumulation of ptox. Previously, we have reported that MeJA significantly induces accumulation of ptox by up regulating the genes in the ptox biosynthetic pathway (Bhattacharyya et al., 2013, 2016). However, the molecular mechanism of MeJA-induced expression of ptox biosynthetic pathway genes is still to be elucidated. In this study ROS has been detected by the FACS and confocal microscopy in MeJA treated *P. hexandrum* cell culture which was simultaneous to the enhanced ptox accumulation (**Figures 1A–C**). Results also indicate the enhanced ptox accumulation in CCCP and H_2_O_2_ treated *P. hexandrum* cell cultures which may be attributed to the enhanced ROS production (**Figures 1A–C**). To further confirm the involvement of ROS in ptox accumulation, in both MeJA and CCCP treated cultures, ROS was inhibited by DPI and DETC and that led to the lower degree of ptox accumulation. This observation confirmed the involvement of MeJA induced ROS in increased accumulation of ptox.

It was reported earlier that ROS plays a significant role as a signaling molecule in plant development, abiotic and biotic stress management, photorespiration and photosynthesis, etc. (Noctor and Foyer, 1998; Apel and Hirt, 2004; Mittler et al., 2004).

To understand how MeJA induced ROS affect the ptox biosynthetic pathway genes consequently up accumulating ptox biosynthesis, the expression pattern of 19 ptox biosynthetic pathway genes was checked at transcript level after the MeJA treatment. Results revealed that all these 19 genes were up regulated after the MeJA treatment. Interestingly, when MeJA induced ROS was inhibited by DPI and DETC treatment, consequently, the up regulation of three genes, namely *PhCAD*3, *PhCAD*4, and *NAC*3 was decreased significantly, whereas other 13 genes were noted with the almost same rate of up regulation as obtained after MeJA treatment without ROS scavenging (**Figure 2A**). Hence, it can be assumed that MeJA induced ROS affect the regulation of *PhCAD*3, *PhCAD*4, and *NAC*3. To confirm the ROS-responsiveness of these three genes, we further treated the culture with CCCP, which induced the ROS generation (Chaudhari et al., 2007; Ji et al., 2016) as well as with H_2_O_2._ Results indicated that only three genes, namely, *PhCAD*3, *PhCAD*4, and *NAC*3, were significantly up regulated after the treatment amongst all 19 genes (**Figures 2B,C**). Furthermore, when ROS generated in CCCP treated culture was inhibited by DPI and DETC treatment, the up regulation of these three genes were deceased significantly. Together, it can be deduced that MeJA induced ROS is the responsible factor for up regulation of these three ROS-responsive genes namely *PhCAD*3*, PhCAD*4, and *NAC*3. In our previous study, we have demonstrated that these two ROS-sensitive isoforms of *PhCAD*, viz. *PhCAD*3 and *PhCAD*4, were specifically responsible for ptox biosynthesis (Bhattacharyya et al., 2016).

We further investigated the underlying mechanism through which ROS-responsive genes of the ptox biosynthetic pathway were regulated by ROS. It is well known that the regulation of mRNA decay is a major checkpoint for controlling the expression level of different genes (Guhaniyogi and Brewer, 2001; Shim and Karin, 2002). According to Chung et al. (2008) the mRNA stability of a particular gene can be altered by ROS. They showed that H_2_O_2_ treatment increases the mRNA stability of the *SOS*1 gene in *A. thaliana*. In our study, the mRNA stability of *PhCAD*3*, PhCAD*4, and *NAC*3 were increased after independent MeJA, CCCP, and H_2_O_2_ treatment. Further, when ROS generated in MeJA and CCCP treated culture, was inhibited by DPI and DETC, the mRNA stability of these three genes was decreased (**Figure 3**). That observation indicates that MeJA induced ROS up regulates *PhCAD*3*, PhCAD*4, and *NAC*3 by increasing their mRNA stability.

We also proposed the possible mechanism of MeJA mediated up regulation of upstream and downstream genes of PhCADs which are not regulated by MeJA produced ROS. According to Carlborg and Haley, (2004) the differential expression of genes can result from *cis*- and/or *trans*-regulatory changes, leading to epistatic effects. miRNA plays a vital role as a *trans*-acting regulator of gene expression. It was previously reported that miR163 plays important role in plant secondary metabolism (Ng et al., 2011). Robert et al. (2011) also reported that miR163 modulates the secondary metabolite pathway from camalexin toward glucosinolates. It was suggested earlier that biosynthesis of benzylisoquinoline alkaloids were might be regulated by pso-miR13, pso-miR2161, and pso-miR408 in *Papaver somniferum* (Boke et al., 2015).

In our study, among eight selected miRNAs, which are targeted to the phenylpropanoid pathway genes, only five miRNAs namely miR172i, miR035, miR1438, miR2275, miR8291 were significantly down regulated after MeJA treatment. Heovere, H_2_O_2_ as well as CCCP induced ROS, did not show any effect on these eight miRNAs (**Figure 4**). Hence, these can be concluded that these five down regulated miRNAs were not regulated by the MeJA induced ROS. These five miRNAs are targeted to four *coumarate ligage* [4*CL*], *Cytochrome P*450, *Caffeoyl–Co*A *methyltransferase*, *Cytochrome P*450, and *C. synthase* as identified by KEGG analysis in our previous report (Bhattacharyya et al., 2016; Biswas et al., 2016). So the down regulation of these ROS non-responsive miRNA in trun bring about the up regulation ptox biosynthetic pathway genes. As we noted here, almost all genes located upstream and downstream of *PhCAD*s, were ROS non-responsive whereas, *PhCAD*s are ROS-responsive. Again, five above mentioned ROS non-responsive miRNAs were also targeted to the genes up- and downstream of *PhCAD*s. These five miRNAs interfere with the target genes expression and the decline in the level of these microRNAs may bring about an up-regulation of the ptox biosynthetic pathway genes. Hence it can be concluded that may be miRNA is just one of the another way to regulate the other sixteen ROS non-responsive genes of the ptox biosynthetic pathway.

## Conclusion

Taken together, it can be concluded that MeJA can up regulate the ptox biosynthetic genes by two possible ways, one through increasing the mRNA stability of *PhCAD*3, *PhCAD*4, and *NAC*3 by MeJA induced ROS and another through the down regulating some miRNAs which are targeted to other important genes of the ptox biosynthetic pathway.

## Author Contributions

SH carried out the experimental work, analyzed the data, and draft the manuscript. DB helps to analyze additional data and performed initial experiments. SC conceived and designed the experiments and prepared the final manuscript.

## Conflict of Interest Statement

The authors declare that the research was conducted in the absence of any commercial or financial relationships that could be construed as a potential conflict of interest.
