# Supplementary material for: Methyl Jasmonate Regulates Podophyllotoxin Accumulation in Podophyllum hexandrum by Altering the ROS-Responsive Podophyllotoxin Pathway Gene Expression Additionally through the Down Regulation of Few Interfering miRNAs
Source: Front Plant Sci. 2017 Feb 14;8:164. doi: 10.3389/fpls.2017.00164 (PMC5306198; doi:10.3389/fpls.2017.00164)
Supplement: Supplementary file 1 [file Table_1.DOC]

| **Supplemental table. 1. Primers for ptox biosynthetic pathway genes and transcription factors** | |
| --- | --- |
| **Group A *PhCAD* isoforms** | |
| **PhCAD1 F** | ATGGTGGCATCTCCAGAG |
| **PhCAD1 R** | 5’ CCCTACCTCAGTCACTAC 3’ |
| **PhCAD2 F** | 5’ AATCAGAGGGAAGTCTCTC 3’ |
| **PhCAD2 R** | 5’ ACTTCCGTCACTACACCAA 3’ |
| **PhCAD3 F** | 5’ AGAGATTCGTCTGGTGTTC 3’ |
| **PhCAD3 R** | 5’ CACCATGCATCCAACACC 3’ |
| **PhCAD4 F** | 5’ ATGTCTGAAGCAATTAAATCAT 3’ |
| **PhCAD4 R** | 5’ TGGTCACTTCGCCGACAA 3’ |
| **Actin F** | 5’ ATCATGAAGTGTGATGTGGAA 3’ |
| **Actin R** | 5’ ATTTAGAAGCACTTCCTGTG 3’ |
| **Group B downstream of *PhCAD*** | |
| **PLR F** | 5’TGGCTAAGAGCAGAGTTC 3’ |
| **PLR R** | 5’CTGCGATGATCATTGAATGA 3’ |
| **DPO F** | 5’ATGGGAGGAGAAAAAGCTTT 3’ |
| **DPO R** | 5’CTTGTGGTGCACCCACAA 3’ |
| **SDR F** | 5’GGCATTTGTGATAGAACCG 3’ |
| **SDR R** | 5’GAACGGAGGACAAGATCG 3**’** |
| **SDG F** | 5’TGAGTGATTCAACGGCTG 3’ |
| **SDG R** | 5’CTCAAATCATCCTCATTAGT 3’ |
| **AdMM F** | 5’GCAACGCTCTGGGATGAA 3’ |
| **AdMM R** | 5’TCACCGACAATCCAGTCC 3’ |
| **Group C upstream of *PhCAD*** | |
| **PAL F** | 5’AGAAGGTGCTCACCATGAA 3’ |
| **PAL R** | 5’TCCTCGAACTTGGTGATCT 3’ |
| **C4H F** | 5’ATCACTCTCTTGCGTTCCT 3’ |
| **C4H R** | 5’CTGATGATGTACAACGACAT 3’ |
| **HCT F** | 5’TACAGAGGACAGCATGGC 3’ |
| **HCT R** | 5’CTCCCTTGGAGTTGGTAT 3’ |
| **CCR F** | 5’GATGAAAACAATCTCAGGCT 3’ |
| **CCR R** | 5’AGCTCGGGTCCACATACA 3’ |
| **4CL F** | 5’AGGATCGCCATCTCCTGA 3’ |
| **4CL R** | 5’GGTTCATCCGCAGCTGCA 3’ |
| **Group D transcription factors related to phenylpropanoid pathway** | |
| **NAC3 F** | 5’TCTGGTATTCTATGCCGGA 3’ |
| **NAC3 R** | 5’GACACGCCATCAGATTCG 3’ |
| **AP2- EREBP8 F** | 5’CTCAAGGTGGAAAGAATATAA 3’ |
| **AP2 –EREBP8 R** | 5’TACCAAACCATAGCCTGCT 3’ |
| **MYB1 F** | 5’ATGCAGCTCGAGTATCTC 3’ |
| **MYB1 R** | 5’CACCTCCATAGATCAATACA 3’ |
| **mTERF1 F** | 5’CAAGCGTCTTTCACCCCA 3’ |
| **mTERF1 R** | 5’TACATGTCCAGGATCCGG 3’ |
| **WRKY71 F** | 5’ACACGTAGTATCTCTTCTCT 3’ |
| **WRKY71 R** | 5’CTGGTGAAAGAAGTCCTC 3’ |
