# Supplementary material for: Methyl Jasmonate Regulates Podophyllotoxin Accumulation in Podophyllum hexandrum by Altering the ROS-Responsive Podophyllotoxin Pathway Gene Expression Additionally through the Down Regulation of Few Interfering miRNAs
Source: Front Plant Sci. 2017 Feb 14;8:164. doi: 10.3389/fpls.2017.00164 (PMC5306198; doi:10.3389/fpls.2017.00164)
Supplement: Supplementary file 2 [file Table_2.DOC]

| **Supplemental table. 2. Primers for miRNAs** | |
| --- | --- |
| **miR1873 SL RT** | 5' – GTCGTATCCAGTGCAGGGTCCGAGGTATTCGCACTGGATACGACCTTCCA- 3' |
| **Forward-miR1873** | 5' - GTTTTCAACATGGTATCAGAGC - 3' |
| **miR2275d SL RT** | 5' – GTCGTATCCAGTGCAGGGTCCGAGGTATTCGCACTGGATACGACCTTGTT - 3' |
| **Forward-miR2275d** | 5' - GTTGGGTGAGATATTGGAGAAA - 3' |
| **miR5532 SL RT** | 5' – GTCGTATCCAGTGCAGGGTCCGAGGTATTCGCACTGGATACGACCCACCT - 3' |
| **Forward -miR5532** | 5' - GTGGGGATGGAATATATGACAA - 3' |
| **miR5538 SL RT** | 5' – GTCGTATCCAGTGCAGGGTCCGAGGTATTCGCACTGGATACGACACTGAA- 3' |
| **Forward-miR5538** | 5' - GTGGCAGCAAGTGATTGAG - 3' |
| **miR829.1 SL RT** | 5' – GTCGTATCCAGTGCAGGGTCCGAGGTATTCGCACTGGATACGACATTCCA - 3' |
| **Forward-miR829.1** | 5' - GTTGAGCTCTGATACCAAATGA - 3' |
| **miR5035 SL RT** | 5' – GTCGTATCCAGTGCAGGGTCCGAGGTATTCGCACTGGATACGACTAAGGG- 3' |
| **Forward-miR5035** | 5'-GGGGGGCTTCTAAACATTTTTT-3' |
| **miR172i SL RT** | 5' -GTCGTATCCAGTGCAGGGTCCGAGGTATTCGCACTGGATACGACAGAATC - 3' |
| **Forward-miR172i** | 5' - GTGGTTGCAGCATCATCAG - 3' |
| **miR1438 SL RT** | 5' –GTCGTATCCAGTGCAGGGTCCGAGGTATTCGCACTGGATACGACAGGGTA - 3' |
| **Forward-miR1438** | 5'-GTTGGGGGGTTAAAAATGATAAAAT-3' |
| **Universal reverse** | 5' - GTGCAGGGTCCGAGGT - 3' |
